# Urinary Deposites, Their Diagnosis, Pathology, and Therapeutical Indications

**Published:** 1845-12

**Authors:** 


*Urinary Deposites, their Diagnosis, Pathology, and Therapeutical indications.* By Golding Bird, A. M., M. D., Assistant Physician to, and Lecturer on Materia Medica at, Guy's Hospital, etc. etc. etc. 8vo. pp. 227. Lea & Blanchard—Philadelphia, 1845.

This is a reprint of the London Edition, without being "*revised with additions*," and is presented to the reader in good clear type and white paper—which is a good deal to say to those whose reading must be accomplished by lamp-light and the aid of "artificial eyes."

Two or three years ago, several lectures on the diagnosis and pathology of urinary sediments, delivered to the pupils of Guy's Hospital by the author, were published in the London Medical

Gazette, and attracted considerable attention at the time by the careful observation and elaborate research which they displayed. Since then, the subject has been further investigated, and is now much extended. The whole subject has been nearly re-written, and the work is placed by the author before the profession as the result of many years' close observation, in the field of public experience which he has had at his command.

Although in this work Dr. Bird has limited his observations to the *deposites* which occur in the urine, and consequently has not included all the conditions of that secretion found in disease, he has nevertheless presented us with a very large amount of useful information on a subject which it is not unjust to say the profession always have been, and probably continue to be, quite uninformed. To those who desire to be better acquainted with the indications of disease presented in the urine, the present volume must prove acceptable.

The body of the work consists of *eleven* chapters, as follows: 1. Physiological origin and physical properties of the urine. 2. Chemical physiology of the urine. 3. Chemical pathology of uric acid and its combinations. 4. Chemical pathology of uric oxide. 5. Chemical pathology of purpurine. 6. Chemical pathology of cystine. 7. Chemical pathology of oxalate of lime, (oxaluria.) 8. Chemical pathology of the earthy salts. 9. Deposits of black or blue colouring matters. 10. General pathology of non-crystalline organic, and organized deposits. 11. Therapeutical employment of remedies influencing the kidneys.

That our readers may be able to judge of the character and style of the work, we shall present a few extracts on points sufficiently isolated and distinct to be understood, although separated from their proper connection.

In his "introductory remarks on the clinical examination of urine," we have the following directions:

"*A.—Urine without any visible deposit.*

A piece of litmus paper should be immersed in the urine, which, if acid, will change the blue colour of the paper to red. Should no change occur, a piece of reddened litmus paper must be dipped in, and if the secretion be alcaline, its blue color will be restored; but if no change occur, the urine is neutral.

Some of the urine should then be gently heated in a polished metallic spoon over a candle, or what is preferable, in a test-tube over a spirit-lamp, and if a white deposit occurs, albumen or earthy phosphates are present; the former, if a drop of nitric acid does not redissolve the deposit, the latter if it does.

If the urine be very highly colored, and undergoes no change by boiling, the colouring matters of bile, blood, or purpurine are present. To determine which, pour a thin layer of urine on the back of a white plate, and allow a few drops of nitric acid to fall in the centre; an immediate and rapidly ending play of colours, from green to red, will occur, if bile, but no such change if purpurine alone exists. Should the highly-coloured urine alter in colour or transparency by heat, the presence of blood must be suspected.

If the addition of nitric acid to deep red urine, unaffected by heat, produces a brown deposit, an excess of uric acid exists. If the urine be pale, immerse the gravimeter, and if the specific gravity be below 1.012, an excess of water exists in the urine, but if above 1.025, the presence of sugar, or excess of urea is indicated. To determine which, place a few drops in a watch-glass, add an equal quantity of nitric acid, and allow the glass to float on some cold water; crystallisation of nitrate of urea will occur in two or three minutes, if the latter exists in excess. Should this change not occur, the urine must be examined specially for sugar, which, it must be remembered, may exist in small quantities, without raising the specific gravity of the fluid.

Should the urine be alcaline, add a drop of nitric acid; if a white deposit occurs, albumen is present; if brisk effervescence follows the addition of the acid, the urea has been converted into carbonate of ammonia.

### *B.—Urine depositing a visible sediment.*

If the deposit is flocculent, easily diffused on agitation, and scanty, not disappearing on the addition of nitric acid, it is chiefly made up of healthy mucus, epithelium, or in women, an admixture of leucorrhœal discharge.

If the deposit is ropy and apparently viscid, add a drop of nitric acid; if it wholly or partly dissolves, it is composed of phosphates, if but slightly affected, of mucus. If the deposit falls like a creamy layer to the bottom of the vessel, the supernatant urine being coagulable by heat, it consists of pus.

If the deposit is white, it consists of urate of ammonia, phosphates, or cystine; the first disappears on heating the urine, the second on the addition of a drop of diluted nitric acid, whilst the third dissolves in ammonia, and the urine generally evolves an odour of sweet-brier.

If the deposit be coloured, it consists of red particles of blood, uric acid, or urate of ammonia, stained with purpurine. If the first, the urine becomes opake by heat; if the second, the deposit is in visible crystals; if the third, the deposit is amorphous, and dissolves on heating the fluid.

Oxalate of lime is often present diffused through urine, without forming a visible deposit; if this be suspected, a drop of the urine examined microscopically will detect the characteristic crystals.

Much of the little time required for the investigation thus sketched out, may be saved by remembering the following facts.

If the deposit be white, and the urine acid, it in the great majority of cases consists of urate of ammonia; but should it not disappear by heat, it is phosphatic.

If a deposit be of any colour inclining to yellow, drab, pink or red, it is almost sure to be urate of ammonia, unless visibly crystalline, in which case it consists of uric acid."

These directions are sufficiently full and precise to aid a beginner most essentially in commencing his observations on this subject.

The first chapter, on the "physiological origin and physical properties of urine," abounds with interesting facts and much ingenious reasoning, but the conclusions to which they arrive will hardly be attained by all who go over the same ground. Our author, without implicitly following in the footsteps of Liebig, will be found quite often enough ready to swear in the language of the master. Sometimes it has seemed to us that the explanations of phenomena by the great chemist are adopted not so much from a conviction of their correctness, as from inability to offer others more satisfactory. In this way the laws of chemistry, as observed in unorganized matter, are allowed to usurp the place of vital actions. We have not the time or the space to enter upon this argument, or to point out the many instances to which we conceive the remarks we have made are applicable. The following observations, however, are not obnoxious to this censure, and are so clear and elementary as to be readily comprehended and appreciated by every one.

"1. In availing himself of the phenomena presented by the urine n disease, it is essential that the practitioner should not fall into the error of regarding a knowledge of the morbid condition of the secretion as alone essential in directing his treatment; nor must he commit the equally serious mistake of regarding every deviation from the natural conditions of the urine as constituting a disease *per se.* The only view that can be legitimately taken of such conditions is to regard them, not as constituting entities of morbid action, but as one of a series of pathological changes going on in the system, and more valuable than others as an index of disease, in consequence of the facility with which it is detected. Hence every abnormal state of the secretion in question should be regarded rather as an indication of some particular phase of morbid action, than as constituting the ailment itself.

It is true, that those pathological states of the urine accompanied by the formation of deposits, or gravel, as they are popularly termed, may, and do, frequently acquire so serious a character as to lead to the formation of the much-dreaded stone or calculus and thus have a claim, from their importance, to be regarded as definite and independent diseases. Still, both in their pathological and therapeutical relations, although frequently compelled, from the irritation they produce, to make the deposits or calculus primary objects of attention, yet we must never lose sight of the fact, that these are but effects not causes; the terminal links in a chain, of which it should be the endeavour of the physician to grasp the beginning.

2. In a physiological sense, the urine must be regarded as arising from three several sources, each acting alike in preserving the equilibrium of the delicately adjusted balance of the secreting functions of the body. The effects of copious aqueous potations in producing a free discharge of pale urine, at once indicates one source of the great bulk of the urinary secretion, and demonstrates one of the most important functions of the kidneys in their pumping off any excess of fluid which may enter the circulation. A second great duty of these organs is shown in the physical and chemical characters of their secretion after the digestion of food is completed. Here it is no uncommon circumstance to detect the presence of some traces of the elements of an imperfectly digested previous meal; and in unhealthy and irritable states of the digestive functions, to discover some abnormal constituent in the urine, arising from the primary malassimilation of the food. Of the former of these states, the peculiar odour and colour of the urine after the ingestion of asparagus and some other bodies affords an example; and a good illustration of the latter condition is met with in the copious elimination of oxalic acid from the blood shortly after a meal in cases of irritative dyspepsia.

Hence the kidneys have the duty of removing from the system any crude or indigested elements of the food which had been absorbed whilst traversing the small intestines and entered the circulating mass; and of excreting the often noxious results of imperfect or unhealthy assimilation. The third function performed by the kidney is its serving as an outlet to evolve from the animal organism those elements of the disorganisation of tissues which cannot serve any ulterior process in the economy, nor be got rid of by the lungs or skin. The disorganisation of tissues here alluded to, is a necessary result of the conditions for the growth and reparation of the body.

3. It is admitted by all, that during each moment of our existence, every atom of the frame is undergoing some change or other; the old matter is absorbed and thrown off by some of the excreting outlets of the body, and new matter is deposited from the blood to supply its place. The old and effete atoms of the animal structure are not excreted in the form of dead tissue, but their elements become re-arranged; one series of combinations thus produced, rich in nitrogen, is excreted by the kidneys, whilst the more highly carbonised products are called upon to perform, chiefly through the medium of the liver, an important office previous to their final elimination.

4. It is therefore necessary to recognise three distinct varieties of the urinary secretion in every case under investigation: Firstly, that passed some little time after drinking freely of fluids, generally pale, and of low specific gravity, (1.003—1.009) *urina potus.* Secondly, that secreted after the digestion of a full meal, varying much in physical characters and of considerable density, (1.020—1.028 or even 1.030,) *urina chyli vel cibi.* Thirdly, that secreted from the blood independently of the immediate stimulus of food and drink, as that passed after a night's rest, *urina sanguinis;* this is usually of average density, (1.015—1.025,) and presents in perfection the essential characters of urine."

In the chapter "on the therapeutical employment of remedies influencing the functions of the kidneys," our author lays down the following laws:

"Law 1st.—All therapeutical agents intended to reach the kidneys must either be in solution when administered, or capable of being dissolved in the fluids contained in the stomach or small intestines, after being swallowed.

"Law 2d.—Bodies intended to reach the kidneys, must, to insure their absorption, have their solutions so diluted as to be of considerably lower density than either the liquor-sanguinis, or serum of blood (i. e. below 1.028.)"

The first law, if law it be, is in accordance with the views we have long entertained and publicly taught, and will hardly be objected to by any—it harmonizes with every day's experience, and is neither novel nor difficult of comprehension. We cannot say, however, that we are prepared to adopt the *second.*

It is confessedly founded on the law of endosmose and exosmose—that double permeation takes place through living as well as dead animal membranes—that the serum of the blood must soak through the blood vessels to be replaced by the penetration of the medicated solution. This may be so, but where is the proof?

From the observations contained in this chapter, Dr. Bird has drawn the following practical conclusions; they seem to us to be ess exceptionable than the source from whence they profess to be deduced.

"1. Whenever it is desirable to impregnate the urine with a salt, or to excite diuresis by a saline combination, it must be exhibited in solution, so diluted as to contain less than five per cent. of the remedy, or not more than about twenty-five grains in an ordinary draught. The absorption of the drug into the capillaries will be ensured by a copious draught of water, or any diluent, immediately after each dose.

2. When the urine contains purpurine, or other evidence of portal obstruction exist, the diuretics or other remedies employed should be preceded or accompanied by the administration of mild mercurials,—taraxacum, hydrochlorate of ammonia, or other cholitic remedies. By these means, or by local depletion, the portal vessels will be unloaded, and a free passage obtained to the general circulation.

3. In cases of valvular or other obstructions existing in the heart and large vessels, it is next to useless to endeavour to excite diuretic action, or appeal to the kidneys by remedies intended to be excreted by them. The best diuretics here will be found in whatever tends to diminish the congested state of the vascular system, and to moderate the action of the heart; as digitalis, colchicum, and other sedatives, with mild mercurials.

